# Analysis of Swallowing Functional Preservation by Surgical Versus CRT After Induction Chemotherapy for Oropharyngeal Cancer

**DOI:** 10.3390/cancers16213658

**Published:** 2024-10-30

**Authors:** Yung-An Tsou, Wen-Dien Chang, Nai-Hsin Meng, Chun-Hung Hua

**Affiliations:** 1 Department of Otorhinolaryngology, China Medical University Hospital, Taichung 404327, Taiwan; entmanhua@gmail.com; 2 School of Medicine, China Medical University, Taichung 406040, Taiwan; d6351@mail.cmuh.org.tw; 3 Department of Audiology and Speech-Language Pathology, Asia University, Taichung 41354, Taiwan; 4 Department of Sport Performance, National Taiwan University of Sport, Taichung 404401, Taiwan; changwendien@ntus.edu.tw; 5 Department of Physical Medicine and Rehabilitation, China Medical University Hospital, Taichung 404327, Taiwan

**Keywords:** oropharyngeal squamous cell cancer, dysphagia, nasogastric tube, chemoradiation, surgery

## Abstract

**Objectives:** This retrospective observational study investigated to determine whether surgery or chemoradiation therapy after induction chemotherapy leads to better swallow function for oropharyngeal cancer patients. **Methods:** We documented the treatment paths and results of 267 patients with oropharyngeal squamous cell cancer (OPSCC). By quantifying nasogastric (NG) tube usage, surgery after induction chemotherapy (IC–surgery), and chemoradiation therapy after induction chemotherapy (IC-CRT) could be compared to determine the effectiveness of each. Cancer stages were also recorded concerning treatment options. The differences in NG tube usage IC–surgery and IC-CRT groups were compared. The NG tube dependence rates were also presented. **Results:** The prognosis and tube dependence differed significantly between the two groups. The IC–surgery had a better prognosis compared to IC-CRT for oropharyngeal cancer. The findings indicated that NG tube dependence was greater in advanced tumor stage 4 compared to stages 1–3, and NG tube dependence rates were higher for patients who underwent chemoradiation therapy after induction chemotherapy. Swallowing function was better in the IC–surgery group compared to the IC-CRT group. **Conclusions:** Higher NG tube retention rates and NG dependence are found in OPSCC patients who choose CRT as their treatment and also in the advanced-stage group.

## Simple Summary

Oropharyngeal squamous cell carcinoma (OPSCC) and its treatments often result in dysphagia, necessitating prolonged use of a nasogastric (NG) tube. NG tube removal rates in patients with OPSCC were compared between two strategies (induction chemotherapy followed by surgery versus induction chemotherapy followed by chemoradiation therapy). The outcomes related to swallowing function and NG tube dependence were shared with clinical physicians for guidance.

## 1. Introduction

Head and neck cancers originate from mucosal surfaces. Approximately 90% of these tumors are squamous cell carcinoma (SCC) [1]. The oropharynx consists of the posterior third of the tongue, the tonsillar fossa, and the soft palate. It also contains the lateral and posterior pharyngeal walls up to the location of the hyoid bone [2]. More than 90% of oropharyngeal squamous cell carcinoma cases are caused by human papillomaviruses (HPV)-16 in HPV-positive SCC [3]. Over recent years, there has been an increase in oropharyngeal cancer among the population aged 20–44 [4]. Carcinoma is affecting young populations at an unprecedented rate across all ethnic groups. SCC incidence has been reduced with a decline in tobacco smoking [5]. Overall, each case of oropharyngeal squamous cell cancer (OPSCC) varies significantly. Factors such as tumor location, size, and treatment (surgery or chemotherapy and radiotherapy) are major contributors to functionality and quality of life post-treatment.

The treatment strategy for oropharyngeal cancer varies according to the patient’s condition, and a tailor-made individualized treatment plan based on the best survival outcomes often compromises the normal function of swallowing and speech [6]. In addition, the disease itself also leads to swallowing, articulation, and even airway dysfunction. Advanced tumor conditions often lead to dysphagia, and deteriorated swallow dysfunction is often noted after treatment via surgery therapy or chemoradiation-initiated therapy [7,8]. According to our literature review, it has been reported that 30–50% of patients need a long-term nasogastric tube, and 10–20% need feeding jejunostomy or gastrostomy [9,10]. Advanced T-stage oropharyngeal cancer leads to a higher rate of nasogastric (NG) and tracheostomy-dependent conditions, and the quality of life is hindered by dysphagia and dyspnea [11,12]. Such conditions are also noted in patients with advanced N-stage oropharyngeal cancer, and dyspnea and airway problems are also affected in patients with advanced TN-stage oropharyngeal cancer [10].

Dysphagia is often found in patients with OPSCC. Early intervention with swallowing and speech therapy is beneficial to patients with oropharyngeal cancers, as it not only leads to better survival outcomes but also to a better quality of life and improved functioning for each patient [13,14]. Clinicians must modify treatment strategies to not only focus on survival outcomes but also quality of life, as good functioning and quality of life can also affect survival outcomes significantly [15].

Most prior studies consider surgery itself to lead to more sequela and push patients to receive chemoradiation therapy [16,17]. However, induction chemotherapy improves the treatment outcomes and leads to more flexible treatment choices for better disease control and quality of life. But there are not many surveys on the prediction of the sequela of dysphagia [18]. OPSCC itself can contribute to various dysphagia conditions, and treatment can lead to different severities of swallowing and respiratory functional loss that not only affect quality of life but also survival rates. Therefore, our study aimed to survey whether surgical or chemoradiation therapy after induction chemotherapy leads to better function, i.e., a decrease in NG tube dependence, and we hope to offer better and proper treatment strategies that lead to not only better survival outcomes but also better functions.

## 2. Materials and Methods

This retrospective study was conducted, and the waiver approval for all clinical data was approved by the Institutional Review Boards of China Medical University Hospital (CMUH110-REC3-037). The cases of OPSCC with tumors on the tongue base, soft palate, and palatine tonsils were included. Exclusion criteria were other head and neck cancers, esophageal cancer, 2nd primary OPSCC, cerebral vessel accident, non-treated palliated OPSCC, and prior oral cavity surgery or radiation. We documented the chart of 267 patients with OPSCC and collected the medical records and use of NG tube after treatments. In the study, the induction chemotherapy is given with biweekly TPF for at least 4 courses. The chemotherapy regimen consisted of docetaxel 50 mg/m^2^ on Day 1, cisplatin 50 mg/m^2^ on Day 1, and 5-fluorouracil 2500 mg/m^2^ administered via continuous infusion over 46 h on Days 1 and 2. The treatment was given every 14 days for up to 4 cycles. After the 4 courses of induction chemotherapy, the patients underwent further chemoradiation or surgery; the decision was non-randomized and made in accordance with patients and their family, head and neck surgeons, medical oncologists, and radiation oncologists after a tumor board meeting. After the treatment, swallowing was considered the functional priority, and the use of an NG tube was examined to compare the effectiveness of surgery and chemoradiotherapy after induction chemotherapy in improving patients’ quality of life. Different stages of cancer were also taken into account in our study to determine the most appropriate treatment options.

Oropharyngeal cancer was categorized as tongue-base cancer, tonsillar cancer, soft palatal cancers, and posterior pharyngeal wall cancers, and the Tumor, Node, and Metastasis (TNM) stage was defined as AJCC 8th edition, guided by extranodal extension (ENE) or not; p16 stain or not means HPV appeared or not. The nodal stage was upgraded if ENE was positive, and the T stage was downgraded if HPV was positive.

### 2.1. Surgery After Induction Chemotherapy (IC–Surgery)

Surgery for OPSCC after IC (induction chemotherapy) varied according to the disease status. Transoral excision by robotic surgery (*n* = 33), transoral laser (*n* = 13), coblation resection (*n* = 28), and Bovie tumor tonsillectomy (*n* = 57) was planned for patients with early-stage oropharyngeal cancers. Mandible swing or maxillectomy, composite resection for advanced oropharyngeal cancer after IC (*n* = 40). Neck dissection was performed for every nodal-positive disease if surgical-initiated therapy was scheduled. Free flap reconstruction or pedicle flap reconstruction, like pectoralis major myocutaneous flap (PMMCF), was performed for advanced defects after tumor resection. No reconstruction was performed for early-stage oropharyngeal cancer patients if no great vessels were threatening in our cohort.

### 2.2. Chemoradiation Therapy After Induction Chemotherapy (IC-CRT)

Radiation therapy combined with chemotherapy was prepared for chemoradiation-initiated therapy in oropharyngeal cancer patients in different disease stages, with 6000–7000 cGy planned for each patient. Cisplatin-based therapy was also planned for each patient in the 1st, 4th, and 7th week during radiation therapy at 100 mg per kilogram. In addition, 5500–6000 cGy chemoradiotherapy (CRT) was planned after induction chemotherapy. Prolonged NG dependence is defined as lasting for more than 6 months.

#### Subgroup Analysis in Advanced Oropharyngeal Cancer

Additionally, we conducted a subgroup survey among patients with an advanced OPSCC cancer stage (stage III-IVa) who were eligible for curative treatment either through IC–surgery or IC-CRT therapy. Among the patients categorized under OPSCC stages III-IVa (n = 89), we examined prolonged NG dependence during the period from 3 to 12 months after treatment, comparing the differences between these two treatment methods. Surgery for advanced OPSCC standardized as fitting disease status, including mandible swing or maxillectomy, and composite resection of oropharyngeal cancer with neck dissection, was performed for every nodal-positive disease if surgical therapy could be performed. Free flap reconstruction was performed for defects after tumor resection.

### 2.3. Statistical Analysis

The frequency distribution and percentage were used to express the categorical variable, while the continuous variable was represented as the mean and standard deviation. The *t*-test and chi-square test were used to compare the patients’ characteristics between the 2 groups. The Cox proportional hazard model was employed to analyze the association between the removal of NG tubes and variables such as age, gender, alcohol, betel nut, cigarette, surgery types, cancer stage, and TNM stage. The Kaplan–Meier method was used to present the NG-tube dependence by log-rank statistics. The contribution of the variables for NG-tube removal was examined using logistic regression. All statistical analyses were performed using SPSS 23.0 (SPSS Inc., Chicago, IL, USA), and statistical significance was set at *p* < 0.05.

## 3. Results

In total, the medical records of 267 OPSCC patients (age = 54.55 ± 9.85) were analyzed. Among the recorded variables, sex, surgery or chemoradiation after induction chemotherapy, and cancer stage showed the effects on the NG removal rate (Table 1). The TNM staging was used to determine the effect on NG dependence among our patients. T refers to the size of the primary tumor, N applies to the number of adjacent lymph nodes with cancer, and M pertains to the metastasis of the tumor. Table 1 compares NG-tube dependence among patients with cancer stages 1–4. Our data indicate that of 201 patients with stages 1–3, only 17 (8.5%) used NG tubes for a prolonged time. The results for stage 4 showed a higher prolonged NG-dependent rate of 30 (45.4%) (NG dependence over 6 months) due to the advancement of the cancer at that point. We noted that age is not a significant factor for NG-tube dependence, but gender is a significant factor for NG-tube dependence. The significant factor is the stage of the cancer, with the T stage and overall stage being correlated with long-term NG-tube dependence. We also found that the NG-tube dependence differed significantly between the IC–surgery and IC-CRT groups. (Table 1).

**Figure 1:**
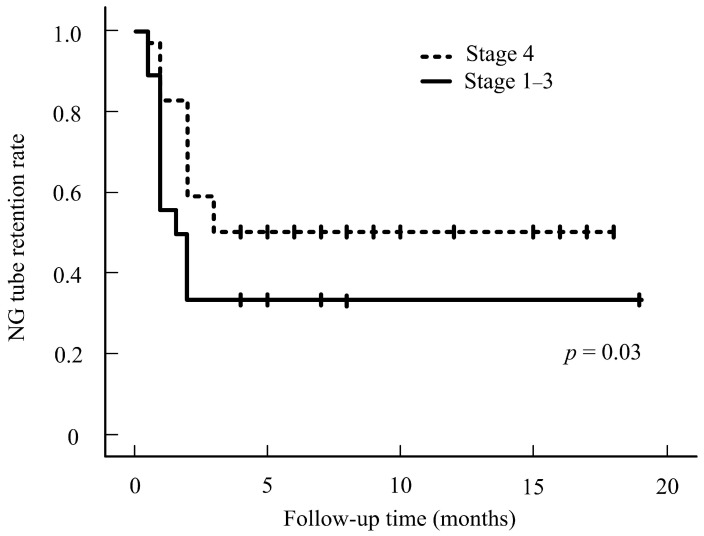
NG rate for stages 1–3 versus stage 4.

**Figure 2:**
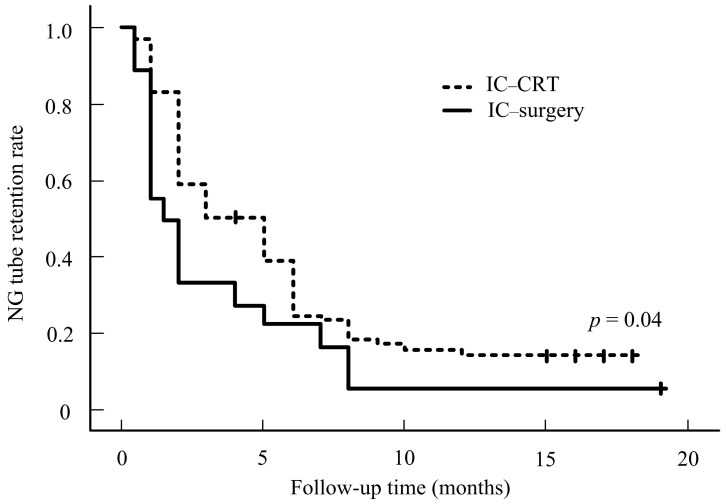
NG rate for IC–surgery vs. IC-CRT.

**Figure 3:**
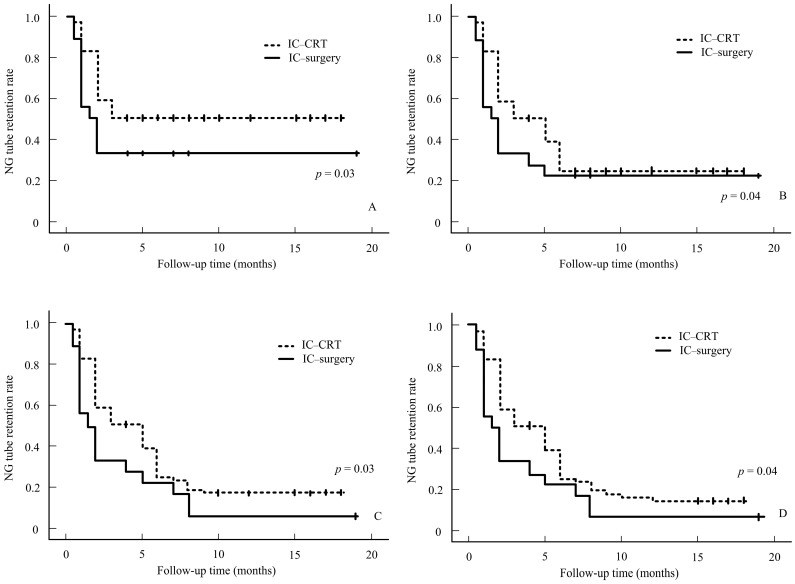
NG rate for IC–surgery vs. IC-CRT at 3 (**A**), 6 (**B**), 9 (**C**), and 12 (**D**) months.

Our results showed that NG-tube dependence is greater in stage 4 than in stages 1–3 (Figure 1). No significant differences in the characteristics were noted in the IC–surgery and IC-CRT groups (all *p* > 0.05, Table 2). Although staging is essential to treat the tumor, we can conclude that the N stage has no significance in regard to NG tube use, as the data collected prove that the N stage is statistically insignificant and is not a factor in tube dependence. Our data suggest that patients who underwent IC-CRT treatment tended to experience a prolonged NG tube-dependence rate compared to those who underwent IC–surgery treatment. This difference in dependence rates is statistically significant. Figure 2 illustrates the NG tube-dependence rate for patients who underwent IC–surgery, indicating a short period of dependency shortly after the IC–surgery. The majority of patients relied on NG tubes for less than a month following the operation in the IC–surgery group. However, our data indicate that prolonged NG-tube dependence can become detrimental over time. In our subgroup analysis of 89 stage III-IVa patients, those who received surgical therapy (n = 40) after induction chemotherapy exhibited a lower NG tube-dependence rate compared to those who underwent IC-CRT (*n* = 49). This trend persisted from 3 to 12 months after treatment, as shown in Figure 3.

## 4. Discussion

HPV has been strongly linked to OPSCC and is considered one of the most potent human carcinogens [19]. The predicted latency period for HPV infection was estimated to be around 20–30 years, suggesting that HPV integration is an early premalignant event. Notably, HPV is integrated in the majority of HPV-positive OPSCC cases, about 50–70%, in the United States [20]. It remains unclear whether HPV integration occurs during the viral life cycle prior to carcinogenesis [20]. However, the majority of HPV-positive OPSCC showed differences in prevalence among Asian populations in our result. The virus produces oncoproteins E6 and E7, which confer it with oncogenic potential through their inhibitory effects on p53 and retinoblastoma (Rb) protein [19]. Patients with a long history of heavy smoking and alcohol consumption also have a higher risk of OPSCC. The incidence of OPSCC has been increasing in younger adults, with the average age ranging from 20 to 44 years old. The previous study showed a particular rise in incidence between the ages of 50 and 59 [19]. However, our study results found that age does not have a significant correlation with NG dependence.

HPV testing is essential in OPSCC due to its role in carcinogenesis. Tumors caused by HPV have a better prognosis and are less likely to recur than tumors not linked to HPV [21]. Stages I and II can be treated with radiation therapy or surgery, and radiation therapy can be administered through intensity-modulated radiotherapy, stereotactic body radiation therapy, or internal radiation therapy. Early stages are presented in a small number of patients and can be treated with surgery or radiation alone [21].

The OPSCC traditionally required invasive surgery that severely reduced the functionality of the organs. However, induction chemotherapy plays a crucial role for treating oropharyngeal cancer. After induction chemotherapy, minimal invasion transoral robotic surgery has emerged as a promising treatment option; chemoradiation therapy, target therapy, or even immune therapy could be performed for OPSCC patients. A small study suggests that transoral robotic-assisted surgery (TORS) has more favorable results in terms of quality of life and functionality compared to primary radiotherapy [22]. These data support a surgical approach, especially for HPV-positive patients who often do not require additional chemoradiotherapy [23]. However, for patients with T3/T4 tumors, treatment options include surgery followed by postoperative irradiation rendered better disease control but higher complications [24]. In recent years, induction chemotherapy has also been crucial for advanced oropharyngeal cancers. Better treatment results and less function sequelae are noted after induction chemotherapy. After induction chemotherapy, surgical consideration is a more promising treatment option than chemoradiotherapy due to the negative impact of dysphagia, morbidity, and a drop in quality of life associated with the latter. However, many patients require a nasogastric (NG) tube throughout their treatment to counteract weight loss. The extended use of an NG tube can cause aspiration pneumonia, gastroesophageal reflux, chronic sinusitis, and lesions to the nasal wing [25]. Furthermore, patients often report discomfort in social activities due to dysphagia, and changing the NG tube due to blockage can be unpleasant in social settings [26,27].

Oropharyngeal cancer itself leads to oropharyngeal dysphagia, oral transient time, pharyngeal transit time, and pharyngeal delay time, all of which increase in advanced tumor stages [28]. Oropharyngeal dysphagia is prominent in these patients before treatment. The post-treatment dysphagia varies in these patients according to the tumor status, treatment strategies, and the patient’s pharyngeal tissue entities. The dysphagia is not limited to the oropharyngeal phase but also extends to the pharyngeal phase and the pharyngeal-esophageal segment after treatment [29,30].

Oropharyngeal cancer patients undergoing chemoradiotherapy (CRT) often experience dysphagia, leading to nutrition problems. Post-radiation laryngeal edema can cause non-functional larynx, dyspnea, and a trachea tube-dependent condition. In contrast, patients undergoing surgical therapy have better overall swallowing-function preservation than those undergoing CRT. This is likely due to better disease control with surgical therapy and the fact that most patients in the surgical group have less radiation toxicity compared to those selected for CRT. Therefore, the size, location, and extent of the tumor are related to the functional outcome in patients with OPSCC [30]. According to Groher et al., if 50% of a structure involved in swallowing is removed, the function will not be seriously impacted [31]. A study by Sessions et al. suggests that the size of the lesion excised is less of a prognostic indicator than the area extended to certain adjacent organs that affect swallowing more. Dysphagia resulting from surgery can be predicted in cases of the base of the tongue and arytenoid cartilage resections [32]. Preservation of the digastric muscle, tongue base, stylohyoid, hypoglossal nerve, and a single set of arytenoids is crucial for swallowing function. Surgical resection of the tumor while preserving these crucial tissues without compromising cancer treatment is considered a better therapy for less recurrent and cancer-resistant conditions. Although the type, location, and size of the tumor play a major role in determining swallowing outcomes, CRT’s effect on swallowing outcomes is detrimental. CRT negatively impacts swallowing function and nutritional intake, and it can cause further tissue damage in the oral cavity, oropharynx, and esophagus, resulting in tissue fibrosis and increased stiffness of the mucosa, leading to dysphagia. However, therapy should be tailored to enhance patients’ quality of life, improve the safety of swallowing and respiration function, reduce dyspnea conditions, and minimize the complications of cancer treatment based on the curative intent of cancer treatment. In our cohort, patient quality of life is better in the surgical group, with efficient management of swallowing function and quicker weaning time of NG tube compared to the CRT group. The tumor and disease stagings between these two groups were insignificant before treatment.

Nonetheless, the stage of cancer remains a crucial factor to consider. The T stage is particularly important during treatment, as it affects the overall quality of life and the dependence on the NG tube. Our results revealed that out of the 201 patients profiled with stages 1–3, 8.7% required NG tubes for an extended period. Our data also indicate that NG-tube dependence is more common in T stage 4 than in T stages 1–3. Staging plays a crucial role in treating OPSCC; however, N stages are not significant factors in the use of NG. The most important finding of this study is that the surgical initiated group had fewer cases of NG-tube dependence. We found that patients with CRT are more likely to require an NG tube.

There were limitations in our cohort. Firstly, we only defined NG dependence as a sequela of dysphagia, but patients with no NG tube did not represent good swallowing function. The independence from the NG tube did not mean that the patient’s swallowing function had recovered to its normal condition. Different degrees of severity of dysphagia may remain, making quantification difficult. In addition, the surgical strategy is still different in early and advanced stages in curative intension, still affecting the swallowing function. Clinical bias arose between the IC + surgery and IC + CRT groups due to the shared decision-making process. Finally, the sample size was small, and the follow-up period was short. A longer follow-up and the study of delayed dysphagia in treated oropharyngeal cancer patients are warranted in the future.

## 5. Conclusions

The group of patients who underwent IC–surgery had better swallowing function compared to those who underwent IC-CRT, as fewer patients required the use of an NG tube in the IC–surgery group. An NG tube is often used to assess functional outcomes and quality of life after treatment; however, patients with no NG tube feeding do not represent good swallowing function. The stage of cancer was found to be statistically significant and a factor in NG tube dependence, but age was not a significant factor. A further mechanical survey of the chemoradiation tissue damage, such as mucositis, fibrosis, and nerve–muscular injury, is warranted for sequela in dysphagia after CRT. Surgical intervention may improve tumor control and ultimately lead to better swallowing and laryngeal function preservation, but it is important to balance organ preservation with curative cancer treatment. Further studies investigating objective swallowing function are recommended.

## Figures and Tables

**Table 1:** Patients’ characteristics and hazard ratio for the variables in NG-tube dependence.

Items	Values (Total *n* =267)	Hazard Ratio (95% CI)	*p*-Value
Age	54.55 ± 9.85	0.98 (0.97~1.02)	0.94
Male sex, no. (%)	254 (95.13%)	0.36 (0.15~0.62)	0.01 *
Alcohol, no. (%)	217 (81.27%)	1.06 (0.64~1.74)	0.81
Betel nut, no. (%)	205 (76.77%)	1.08 (0.68~1.71)	0.73
Cigarette, no. (%)	234 (87.64%)	1.26 (0.71~2.23)	0.42
HPV-positive (%)	81 (30.34%)	0.54 (0.21~0.78)	0.65
IC–surgery	171 (64.04%)	0.23 (0.12~0.42)	0.01 *
IC-CRT	96 (35.95%)	2.77 (1.82~4.22)	0.01 *
Cancer stage			
Stage 1–3 vs. 4, no.	201/66	0.53 (0.27~0.96)	0.03 *
TNM stage			
T stage (T0–2) vs. (T3–4), no.	186/81	3.04 (2.01~4.60)	0.01 *
N stage (N0–1) vs. (N2–3), no.	119/148	0.78 (0.51~1.18)	0.24
M stage (M0) vs. (M1), no.	258/9	0.33 (0.04~2.42)	0.28

* *p* < 0.05. HPV, human papillomaviruses; IC, induction chemotherapy; OP, operation; CRT, chemoradiotherapy; TNM, Tumor, Node, and Metastasis.

**Table 2:** Patients’ characteristics in IC–surgery and IC-CRT groups.

Items	IC–Surgery (*n* =171)	IC-CRT (*n* = 96)	*p*-Value
Age	54.82 ± 9.81	53.98 ± 9.89	0.50
Male sex, no. (%)	167 (97.66%)	87 (90.62%)	
Alcohol, no. (%)	141 (82.45%)	76 (79.16%)	
Betel nut, no. (%)	130 (76.02%)	75 (78.12%)	
Cigarette, no. (%)	149 (87.13%)	85 (88.54%)	
HPV-positive (%)	51 (29.82%)	30 (31.25%)	
Cancer stage			
Stage 1–3 vs. 4, no.	36/135	30/66	0.06
TNM stage			
T stage (T0–2) vs. (T3–4), no.	118/53	67/29	0.89
N stage (N0–1) vs. (N 2–3), no.	84/87	35/61	0.05
M stage (M0) vs. (M1), no.	171/0	87/9	N.S.
Overall survival Stages 1–2 Stages 3–4	73% 51%	75% 48%	0.73
Tumor location			
Tonsil	35 (20.36%)	16 (16.67%)	
Tongue base	43 (25.14%)	29 (30.20%)	
Tonsil + tongue base + adjacent tissue	90 (52.63%)	51 (53.12%)	

IC, induction chemotherapy; CRT, chemoradiotherapy; TNM, Tumor, Node, and Metastasis.

## Data Availability

Data are contained within the article.
